# Classification of long-term care wards and their functional characteristics: analysis of national hospital data in Japan

**DOI:** 10.1186/s12913-018-3468-0

**Published:** 2018-08-22

**Authors:** Ayumi Igarashi, Noriko Yamamoto-Mitani, Kojiro Morita, Hiroki Matsui, Claudia K.Y. Lai, Hideo Yasunaga

**Affiliations:** 1 Department of Gerontological Home Care and Long-term Care Nursing, School of Health Sciences & Nursing, Graduate School of Medicine, The University of Tokyo, 7-3-1 Hongo, Bunkyo-ku, Tokyo, 113-0033 Japan; 2 Department of Clinical Epidemiology and Health Economics, School of Public Health, The University of Tokyo, 7-3-1 Hongo, Bunkyo-ku, Tokyo, 113-0033 Japan; 3 School of Nursing, The Hong Kong Polytechnic University, Yuk Choi Road, Hung Hom, Hong Kong SAR

**Keywords:** Cluster analysis, Healthcare policy, Healthcare reform, Hospital, Long-term care ward

## Abstract

**Background:**

In a rapidly aging society that has promoted extensive reforms of the healthcare system, clarifying functional patterns in long-term care wards is important for developing regional healthcare policies. This study aimed to classify patterns of inpatient characteristics among Japanese long-term care wards and to examine hospital/ward characteristics.

**Methods:**

We analyzed data from 1856 long-term care wards extracted from the 2014 Annual Report for Functions of Medical Institutions in Japan. We classified five clusters of long-term care wards based on inpatients’ medical acuity/activities of daily living using cluster analysis, and compared hospital/ward characteristics across the clusters with a chi-square test or analyses of variance.

**Results:**

Cluster 1 was low medical acuity/high activities of daily living (*n* = 175); cluster 2, medium medical acuity/high activities of daily living (*n* = 340); cluster 3, medium medical acuity/low activities of daily living (*n* = 461); cluster 4, high medical acuity/low activities of daily living (*n* = 409); and cluster 5, mixed (*n* = 471). Although clusters 1 and 2 had similar higher proportions of home discharge (48.1% and 34.6%, respectively), there was a difference in length of hospital stay between the clusters (154.6 and 216.6 days, respectively). On the other hand, clusters 3 and 4 experienced a longer length of hospital stay (295.7 and 239.8 days, respectively) and a higher proportion of in-hospital deaths (42.7% and 50.2%, respectively). Characteristics of cluster 5 were not significantly different from the average of overall wards.

**Conclusions:**

There were distinctive differences across hospitals in their use of long-term care wards. Wards with different functions have different support needs; the clusters with high activities of daily living needed support in promoting home discharge, while those with low activities of daily living needed support in providing quality end-of-life care. Our results can be useful for constructing the future regional healthcare system. This study also suggests introducing a standardized patient classification system in long-term care settings.

## Background

In the rapidly aging society in Japan, the government has promoted extensive reform to the healthcare system to control healthcare expenditure [1, 2]; a community-based integrated care system has been developed with functional differentiation among various healthcare facilities [3]. Among the healthcare facilities, long-term care (LTC) wards have an important role for community-based integrated care systems. LTC wards provide long-term care for older adults with severe physical and cognitive problems under the national healthcare insurance system [4]. Patients in LTC wards are generally admitted from acute/subacute hospital wards after acute treatments, or from home due to exacerbation of their disease conditions.

Japanese LTC wards are comparable to skilled nursing homes in Western countries. Since nursing homes in Japan covered by LTC insurance provide limited medical care for older adults due to the small number of healthcare professionals such as physicians and nurses, under the regulation, older patients in need of medical care are admitted to the LTC wards covered by national healthcare insurance. On the other hand, LTC wards also admit some older patients for non-medical reasons. Since patients’ medical needs and activities of daily living (ADL) vary greatly, healthcare reform is required.

Under the healthcare reform of the past decade, the Japanese government began urging municipalities to reconstruct their regional healthcare system based on data, which includes the control of the number of regional hospital beds [5], and introduced the “Annual Report for Functions of Medical Institutions” system in 2014 [6] to obtain effective data for the reconstruction. For the report, all healthcare facilities must submit information regarding hospital beds/staffing and medical contents provided in their facility to the prefectures every year. This new system creates a data-based regional healthcare system for what type and how many healthcare resources are needed, and what functions each healthcare facility should play in the region.

The Japanese government is planning to convert LTC wards in hospitals to non-medical LTC facilities as a part of the healthcare reform. However, a better understanding of the functions of LTC wards is crucial to determine how existing LTC beds should be used in a future healthcare system. Recently, indicators for the functions of acute care hospitals have been developed based on the Annual Report for Functions of Medical Institutions data [7, 8]; however, the functions of LTC wards have yet to be fully examined.

This study aimed to identify patterns of inpatient characteristics among the LTC wards in Japan and to examine the hospital/ward characteristics across these patterns, using the data from the Annual Report for Functions of Medical Institutions.

## Methods

### Payment system by case-mix classification

In the Japanese LTC wards, fees for medical treatments are paid based on a case-mix classification system. The case-mix classification is defined by a combination of medical acuity levels and ADL functional scores: a 3 × 3 matrix with three levels of medical acuity and three levels of ADL. The government determined the tariffs for each group based on the costs of providing standardized services [9]. Under the national healthcare insurance system in Japan, patients are responsible for paying 10–30% of the tariffs, while their insurance agency (municipalities) pays the remaining cost to the hospital.

Classification criteria have been determined for medical acuity and ADL level, respectively. For medical acuity, Level 3 is the status requiring 24-h monitoring by physicians and nurses, including subacute myelo-optic neuropathy (SMON), total parenteral nutrition, being on a medical ventilator, drainage, tracheotomy care with fever, and oxygen therapy. Level 2 includes: multiple sclerosis, neurological disease, Parkinson’s disease, spinal injury with paraplegia, emphysema (COPD), cancer requiring pain control, pneumonia, urinary tract infection, wound infection, persistent vomiting, pressure ulcer, delirium, depression, violent behavior, dialysis, tube feeding with fever, aspiration (eight or more times per day), tracheotomy care, blood sugar check (three or more times per day), and foot care. Level 1 includes conditions other than Level 2 and 3. ADL levels are determined by the summation of the ADL scores for ADL activities of bed mobility, transfer, eating, and toileting, each measured on a scale of 0 to 6 (higher scores = more dependent). Level 3 corresponds to scores of 23−24 point, Level 2 to 11−22, and Level 1 to 0−10 [9].

We used medical acuity and ADL to determine the characteristics of inpatients in LTC wards.

### Subjects

For this study, we obtained data from the 2014 Annual Report for Functions of Medical Institutions from the Ministry of Health, Labour and Welfare, which was the latest issue when the study was initiated. The same data are publicly available from the different prefecture websites [6].

The report consists of hospital and ward data. Hospital data includes characteristics of hospitals (e.g., the number of beds and types of wards) and the contents of healthcare delivery such as information on case-mix classifications of inpatients. Ward data include the characteristics of wards such as bed functions and staffing. Fees for hospitalization in LTC wards were classified into two types based on nurse-to-patient ratio; type-1 fees are paid to the LTC wards with nurse-to-patient ratios of > 1:20, while type-2 fees are paid to LTC wards with nurse-to-patient ratios of 1:25 to 1:20.

**Fig. 1:**
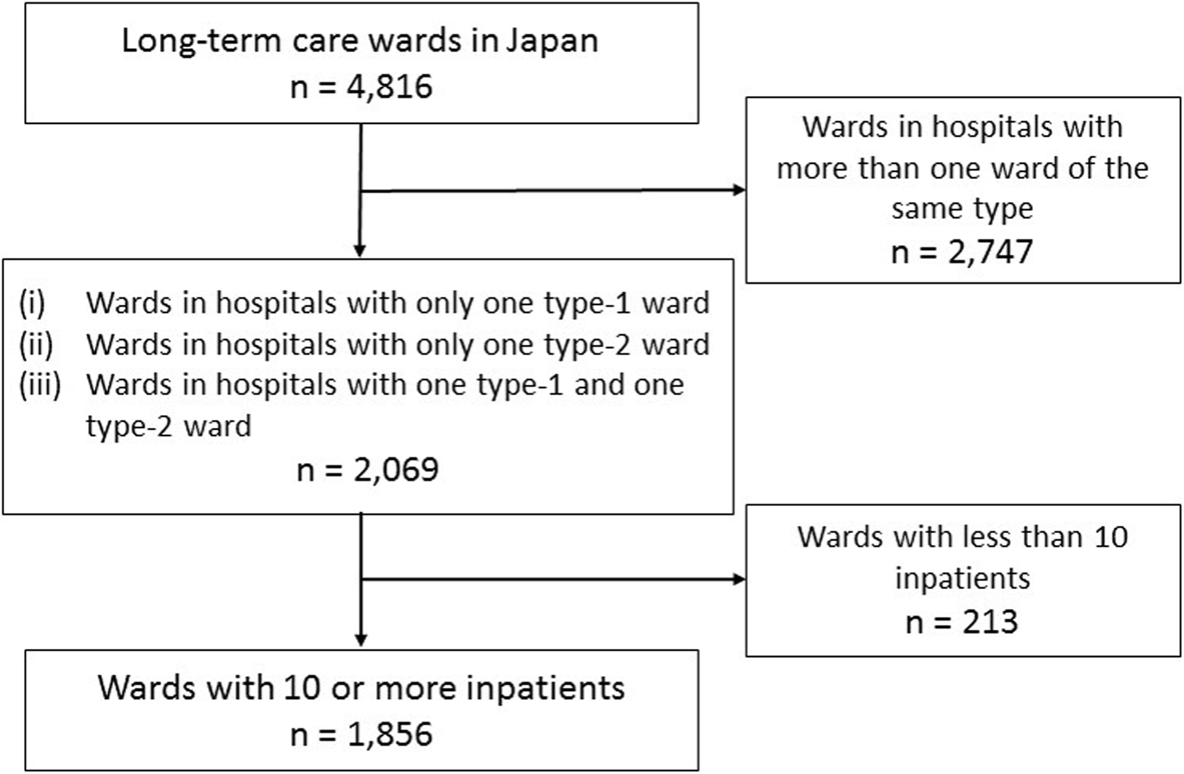
Participant flow

In the Annual Report for Functions of Medical Institutions, data on case-mix classifications were reported by combining all wards of the same fee type: if a hospital had two or more wards of the same fee type (type-1 or type-2), we could not specify the case-mix classifications of each ward. For this study, therefore, we included (i) hospitals with only one type-1 ward, (ii) hospitals with only one type-2 ward and (iii) hospitals with one type-1 and one type-2 ward, and excluded hospitals with more than one type-1 and/or one type-2 ward. Using these criteria, we selected 2069 wards from 4816. Next, we selected 1856 wards to which 10 or more inpatients were admitted during the study period (Fig. 1).

### Measures

We used data on the characteristics of hospitals and wards and the case-mix classifications of inpatients. The characteristics of the hospitals included: the number of beds, whether the hospital had acute care wards, sub-acute care wards, rehabilitation wards, and community-based integrated care wards. The characteristics of the wards included: the number of beds; the average length of hospital stay; bed occupancy rates; nurse-to-patient ratio; the number of full-time equivalent nurses and care workers per 100 beds; the number of patients who were admitted from other wards in the same hospitals, other hospitals, non-medical LTC facilities, or home; the number of patients who were discharged to other wards in the same hospital, other hospitals, non-medical LTC facilities, or home; and the number of in-hospital deaths in June 2014. We used the nine categories of the case-mix classification fees (the 3 × 3 matrix of medical acuity and ADL classifications, please see Table 2) to calculate patient characteristics.

The bed occupancy rate and the average inpatient days in each ward were calculated as follows:

$$ \mathrm{bed}\ \mathrm{occupancy}\ \mathrm{rate}=\frac{\mathrm{total}\ \mathrm{in}\mathrm{patient}\ \mathrm{days}\ \mathrm{in}\ \mathrm{the}\ \mathrm{year}\ }{\mathrm{the}\ \mathrm{number}\ \mathrm{of}\ \mathrm{bed}\mathrm{s}\ \mathrm{in}\ \mathrm{the}\ \mathrm{wards}\times 365\ } $$

$$ \mathrm{the}\ \mathrm{average}\ \mathrm{in}\mathrm{patient}\ \mathrm{days}=\frac{\mathrm{total}\ \mathrm{in}\mathrm{patient}\ \mathrm{days}}{\left(\mathrm{no}.\mathrm{of}\ \mathrm{patients}\ \mathrm{admitted}\ \mathrm{in}\ \mathrm{the}\ \mathrm{last}\ \mathrm{year}+\mathrm{no}.\mathrm{of}\ \mathrm{patients}\ \mathrm{discharged}\ \mathrm{in}\ \mathrm{the}\ \mathrm{last}\ \mathrm{year}\right)\times 1/2} $$

In order to eliminate the impact of outliers, we deleted values < 5 and > 95 percentiles and replaced them with missing values, because such data could not be realistically correct (e.g., number of admissions). The criteria were determined after examining the distribution of each variable.

### Ethical consideration

This study was based on secondary analyses of publicly available data. The data did not include individual-level patient data. We did not obtain written consent from the authorities in each hospital, since the data is publicly available on the prefectures’ websites.

### Statistical analysis

Descriptive analyses were conducted for the case-mix classifications in the LTC wards and the hospital/ward characteristics. We conducted a cluster analysis based on the percentage of patients categorized for each of the nine case-mix classifications. The results from five clusters had the best conceptual fit. We compared the characteristics of hospitals/wards among the five clusters with chi square tests or analyses of variance. For all analyses, SPSS version 22.0 was used (IBM Corp., 2013). The significance level was set at < 0.05 (two-tailed).

## Results

### Characteristics of hospitals/wards

Table 1 shows the hospital/ward characteristics. The average number of all beds ± standard deviation (SD) in the hospitals was 121.9 ± 81.0, and in the LTC wards was 45.1 ± 13.0. The average length of hospital stay was 240.2 ± 144.0 days and the average bed occupancy rate was 89.2 ± 13.5%. Approximately 60% of the wards had nurse-to-patient ratios of less than 1:20. The average number of full-time equivalent nurses (including registered nurses and licensed practical nurses) per 100 beds ± SD was 30.9 ± 9.7, and the average number of full-time equivalent care workers was 26.4 ± 8.4.

**Table 1:** Characteristics of hospital and long-term care wards in each cluster

	All n = 1856	Low medical acuity/high ADL *n* = 175	Medium medical acuity/high ADL *n* = 340	Medium medical acuity/low ADL *n* = 461	High medical acuity/low ADL *n* = 409	Mixed *n* = 471	*p*-value
	Mean ± SD or n (%)	Mean ± SD or n (%)	Mean ± SD or n (%)	Mean ± SD or n (%)	Mean ± SD or n (%)	Mean ± SD or n (%)	
Characteristics of hospital							
Number of beds	121.9 ± 81.0	124.5 ± 98.5	109.5 ± 77.6	119.1 ± 77.2	118.3 ± 68.4	135.8 ± 88.0	< 0.001^†^
Other wards in the hospital							
General ward	1121 (60.4%)	102 (58.3%)	200 (58.8%)	242 (52.5%)	289 (70.7%)	288 (61.1%)	< 0.001^‡^
Sub-acute ward	263 (14.2%)	20 (11.4%)	47 (13.8%)	60 (13.0%)	75 (18.3%)	61 (13.0%)	< 0.001^‡^
Rehabilitation ward	372 (20.0%)	36 (20.6%)	61 (17.9%)	108 (23.4%)	79 (19.3%)	88 (18.7%)	< 0.001^‡^
Community integrated care ward	46 (2.5%)	0 (0%)	8 (2.4%)	13 (2.8%)	15 (3.7%)	10 (2.1%)	< 0.001^‡^
Characteristics of ward							
Number of beds	45.1 ± 13.0	40.9 ± 11.2	43.7 ± 11.8	45.1 ± 15.5	45.3 ± 12.3	47.5 ± 11.9	< 0.001^†^
Average length of stay	240.2 ± 144.0	154.6 ± 107.3	216.6 ± 138.1	295.7 ± 152.6	239.8 ± 128.9	229.1 ± 142.2	< 0.001^†^
Bed occupancy rate (%)	89.2 ± 13.5	83.9 ± 12.8	88.4 ± 13.6	91.5 ± 13.0	91.8 ± 13.1	87.4 ± 13.6	< 0.001^†^
Hospitalization basic rate							
I (20:1 of patient to nurse ratio)	1087 (58.6%)	6 (3.4%)	275 (80.9%)	383 (83.1%)	351 (85.8%)	72 (15.3%)	< 0.001^‡^
II (25:1 of patient to nurse ratio)	769 (41.4%)	169 (96.6%)	65 (19.1%)	78 (16.9%)	58 (14.2%)	399 (84.7%)	
Number of FTE nurses	30.9 ± 9.7	31.0 ± 12.0	32.4 ± 12.6	31.4 ± 8.4	32.0 ± 8.3	28.4 ± 7.9	< 0.001^†^
Number of FTE care workers	26.4 ± 8.4	25.5 ± 9.1	26.3 ± 8.3	27.4 ± 8.1	26.7 ± 8.6	25.4 ± 8.1	0.002^†^
Number of admitted patients in a month	8.5 ± 13.5	13.6 ± 15.6	10.1 ± 18.8	6.0 ± 8.0	8.4 ± 14.6	8.0 ± 10.7	< 0.001^†^
Place before admission							
Other ward in same hospital (%)	59.3 ± 44.0	56.3 ± 44.4	54.2 ± 45.2	51.5 ± 44.7	70.5 ± 40.7	62.1 ± 43.0	< 0.001^†^
Other hospital (%)	21.7 ± 31.7	17.5 ± 27.5	19.2 ± 27.5	28.4 ± 35.5	19.0 ± 31.6	20.9 ± 31.4	< 0.001^†^
Long-term care facility (%)	4.9 ± 13.8	3.0 ± 8.4	4.8 ± 13.2	6.2 ± 17.3	3.9 ± 11.6	5.1 ± 13.9	0.054^†^
Home (%)	13.8 ± 24.0	22.9 ± 30.7	21.6 ± 29.5	13.3 ± 23.2	6.4 ± 15.4	11.4 ± 20.9	< 0.001^†^
Other (%)	0.3 ± 3.4	0.2 ± 2.6	0.2 ± 2.8	0.4 ± 4.2	0.1 ± 1.2	0.6 ± 4.6	0.309^†^
Number of discharged patients in a month	8.2 ± 10.7	13.1 ± 10.7	9.9 ± 16.8	5.8 ± 5.5	7.8 ± 11.6	8.0 ± 6.7	< 0.001^†^
Place after discharge							
Other ward in same hospital (%)	14.2 ± 23.4	10.4 ± 18.2	16.0 ± 25.1	15.3 ± 25.5	12.8 ± 22.2	14.6 ± 22.6	0.064^†^
Other hospital (%)	11.8 ± 19.7	10.4 ± 15.3	13.0 ± 19.8	14.7 ± 24.5	8.7 ± 16.7	11.5 ± 17.7	< 0.001^†^
Long-term care facility (%)	14.5 ± 18.9	18.0 ± 18.2	12.9 ± 17.6	10.8 ± 19.8	12.6 ± 17.4	19.4 ± 21.1	< 0.001^†^
Home (%)	23.9 ± 26.7	48.1 ± 28.2	34.6 ± 29.5	16.1 ± 22.6	15.2 ± 20.7	22.2 ± 24.1	< 0.001^†^
Death (%)	34.9 ± 31.3	12.0 ± 20.8	22.4 ± 25.5	42.7 ± 33.9	50.2 ± 29.9	31.6 ± 27.4	< 0.001^†^
Other (%)	0.7 ± 5.7	1.0 ± 5.6	1.1 ± 8.6	0.5 ± 5.2	0.5 ± 3.7	0.6 ± 4.9	0.510^†^

*SD* standard deviation, *FTE* full time equivalent

Percentages for each item were calculated after excluding missing values

†analysis of variance (ANOVA); ‡ chi-squared test

Among the participating LTC wards, the proportions of admissions from other wards in the same hospital, from other hospitals, and from home were 59.3%, 21.7%, and 13.8%, respectively. In-hospital mortality was 34.9%.

### Clusters of LTC inpatients

Using cluster analysis, we divided case-mix classification patterns into five clusters (Table 2). We named each cluster according to the medical acuity and ADL patient characteristics: cluster 1, low medical acuity/high ADL (*n* = 175, 9.4%); cluster 2, medium medical acuity/high ADL (*n* = 340, 18.3%); cluster 3, medium medical acuity/low ADL (*n* = 461, 24.8%); cluster 4, high medical acuity/low ADL (*n* = 409, 22.0%); and cluster 5, mixed (*n* = 471, 25.3%).

**Table 2:** Percentage of the case-mix classifications in each cluster

All wards (*n* = 1856)				
		Medical acuity level		
		1	2	3
A D L	1	6.9%	7.5%	2.1%
	2	7.7%	11.9%	5.3%
	3	8.3%	23.9%	26.5%
1) Low MA/high ADL (n = 175)				
A D L	1	**31.0%**	14.3%	2.8%
		**(24.1%)**	(6.9%)	(0.7%)
	2	15.1%	9.6%	3.7%
		(7.4%)	(−2.4%)	(−1.6%)
	3	9.2%	7.4%	7.0%
		(0.9%)	(−16.5%)	(− 19.5%)
2) Medium MA/high ADL (n = 340)				
A D L	1	4.9%	**18.9%**	2.5%
		(−2.0%)	**(11.4%)**	(0.4%)
	2	5.0%	**25.2%**	6.8%
		(−2.7%)	**(13.3%)**	(1.5%)
	3	2.7%	18.7%	15.2%
		(−5.6%)	(−5.2%)	(−11.3%)
3) Medium MA/low ADL (n = 461)				
A D L	1	2.2%	3.4%	1.3%
		(−4.7%)	(−4.1%)	(−0.8%)
	2	3.6%	10.0%	3.5%
		(−4.0%)	(−1.9%)	(−1.8%)
	3	6.3%	**44.3%**	25.3%
		(−1.9%)	**(20.4%)**	(−1.2%)
4) High MA/low ADL (n = 409)				
A D L	1	2.7%	3.4%	2.8%
		(−4.2%)	(−4.1%)	(0.8%)
	2	3.3%	6.2%	7.2%
		(−4.3%)	(−5.7%)	(1.9%)
	3	4.3%	16.3%	**53.7%**
		(−4.0%)	(−7.6%)	**(27.2%)**
5) Mixed (n = 471)				
A D L	1	7.6%	4.2%	1.6%
		(0.7%)	(−3.2%)	(−0.5%)
	2	14.5%	10.0%	4.9%
		(6.9%)	(−1.9%)	(−0.4%)
	3	17.3%	20.4%	19.4%
		(9.0%)	(−3.5%)	(−7.1%)

*MA* medical acuity

Percentages in each table indicate the proportion among the inpatients, and (%) indicates differences with those in overall average

Bold data are a feature of each cluster

Cluster 1 was the group that had the highest proportion of patients with low medical acuity and high ADL, making up only 9.4% of the all wards. Among the five clusters, this group had the shortest average length of hospital stay (154.6 ± 107.3 compared to 240.2 ± 144.0 of the overall mean), and the lowest average bed occupancy rate (83.9 ± 12.8%). Cluster 1 had the largest number of admissions and discharges in a month (13.6 and 13.1 patients, respectively), and the proportions of admissions from home and discharge to home were the highest (22.9% and 48.1%, respectively). Although almost all wards in this cluster had nurse-to-patient ratios of about 1:25, the actual number of nurses and care workers per 100 beds were similar to those in other clusters with nurse-to-patient ratios of less than 1:20.

Cluster 2 had a high proportion of patients with medium medical acuity and high ADL, making up 18.3% of the wards. In this cluster, while the proportions of admissions from home and discharge to home were higher (21.6% and 34.6%, respectively), the average length of hospital stay was not short (216.6 ± 138.1 days) and the majority (80.9%) had a nurse-to-patient ratio of less than 1:20.

Cluster 3 had a high proportion of patients with medium medical acuity and low ADL, making up 24.8% of all wards. In this cluster, the average length of hospital stay was the longest (295.7 ± 152.6 days); the admissions from other hospitals/clinics (28.4%), and in-hospital mortality (42.7%) were higher than in the previously mentioned clusters.

Cluster 4 was the group that had a high proportion of patients with high medical acuity and low ADL, making up 22.0% of the all wards. Approximately 70% of hospitals with wards from this cluster had general wards, and the average length of hospital stay was 239.8 ± 128.9 days. In this cluster, the admissions from other wards in the same hospital (70.5%), and in-hospital mortality (50.2%) were highest in this cluster.

Cluster 5 was the group that had a mixed distribution similar to the total sample with a relatively higher proportion of patients with low medical acuity and ADL. This cluster makes up 25.3% of the all wards. While the majority of wards (84.7%) in this cluster had a nurse-to-patient ratio of about 1:25, other characteristics of hospitals/wards were not significantly different from the average of the overall wards.

## Discussion

In this study, we examined the patterns of the patient case-mix classifications via medical acuity and ADL levels in LTC wards in Japan, and clarified the characteristics of hospitals and wards in each classification. To our knowledge, this is the first study to classify LTC wards with a statistical approach, using national data. The methodology and results of this study can be used by local municipalities and healthcare facilities to reconstruct the regional healthcare system, and by the government to develop future healthcare policies.

First, we identified two types of clusters that had large proportions of patients with a high level of ADL: “low medical acuity/high ADL” and “medium medical acuity/high ADL.” We found common characteristics of functions between the two clusters: a high turnover of patients and a relatively large proportion of patients discharged home (48.1% and 34.6%, respectively), suggesting that the clusters support patients in returning to their home after a relatively short treatment and rehabilitation period.

The functions of the “medium medical acuity/high ADL” cluster should have been recognized in healthcare reform. The additional payment to increase discharge home was introduced in 2014 for the LTC wards with a nurse-to-patient ratio of 1:20, and more than 50% of patients were discharged [10]. The majority of this cluster may have already been supported by this additional payment because most had high nurse-to-patient ratios and a high proportion of patients were discharged.

Moreover, the “low medical acuity/high ADL” cluster could also play an important role in treatment and rehabilitation aiming to discharge patients with low medical acuity. This cluster had a similar staffing level with a nurse-to-patient ratio of 1:20. However, in Japanese healthcare policy, the wards in this cluster are not supported by the additional payment for promoting discharge home because of the low medical acuity level of inpatients. Furthermore, despite a need for healthcare workers’ resources, this cluster could be the target for a reduction of hospital beds in the healthcare reform (i.e., changing to non-medical LTC facilities), because of their low medical acuity. The government should recognize this cluster’s function and introduce a special care delivery system to further enhance discharging of patients back home, such as assuring a sufficient number of nurses and care-workers conduct effective rehabilitation and discharge coordination.

Second, in the clusters of “medium medical acuity/low ADL” and “high medical acuity/low ADL,” large proportions of patients died in hospital (42.7% and 50.2%, respectively) after longer stays, whereas the patients in the “medium medical acuity/high ADL” cluster were more likely to be discharged. In addition to suggestions that low ADL was a barrier for institutionalized patients to be discharged in previous studies [11, 12], the need for medical care would make it more difficult to discharge patients. Therefore, these types of wards could play an important function as places to live until death, as well as providing medical care; our results support the findings that this type of ward should also be maintained in the healthcare reform. Further, among all the clusters, patients’ quality of life, especially at the end of life, would be more important in these clusters. End-of-life care education for nurses and care workers in the ward may be effective in improving care quality including control of symptoms, possibly leading to a higher patient quality of life [13].

Finally, in the “mixed” cluster pattern, staff provided care to inpatients with a variety of conditions; the length of stay in the hospital was relatively long, and in-hospital mortality was higher than the proportion of patients discharged. The wards in this cluster also double-up as places to live for patients with medical care needs but who have relatively low acuity, including end-of-life care. This need in a LTC hospital may be due to a lack of community healthcare/long-term care resources in certain regions [14, 15] and of informal care [16] (i.e., social hospitalization). While this cluster should be targeted for bed reductions in the healthcare reform because of the low medical acuity, the national and municipal government should consider the actual situation of healthcare resources in each region and their cost [15], and should make an effort to increase healthcare resources in the community if required. Simultaneously, the results may show that this type of LTC ward is necessary, even with sufficient community resources, depending on the family resources of each patient.

These findings have important implications for LTC policy worldwide; types of facilities and nurse/care worker staffing levels, including payment systems in LTC, must be determined based on an appropriate patient classification system. This study revealed that there were various types of medical LTC wards for inpatients in Japan. Fee schedules for LTC wards in hospitals are classified into nine categories based on medical acuity and ADL levels, while fees for home care or non-medical LTC facilities are paid via LTC insurance based on certified-care need levels [17]. Future healthcare reform should be organized so that the amount of care needed and the staffing levels are measured consistently using a standardized patient classification system, such as the Resource Utilization Groups (RUG-III) [18, 19], regardless of the types of facilities in the LTC settings.

The present study has several limitations. First, the analysis of this study was ward-based; hence the conditions of individual patients were unknown. Second, the Annual Report for Functions of Medical Institutions data is self-reported by each hospital and the reporting system only started in 2014, therefore, the reliability of the data were also uncertain. To minimize the unreliability, we replaced extreme values with missing values. Finally, since we used data from only selected hospitals, there may be a selection bias.

## Conclusions

Despite these limitations, we identified patterns of case-mix classifications in Japanese LTC wards. The results indicated that each pattern has specific characteristics, and each ward plays a different function and has different support needs. This will be useful for constructing future regional healthcare systems from the perspective of the national government, local municipalities, and healthcare facilities. Furthermore, this study also suggests introducing standardized patient classification systems consistently in LTC settings.

## Abbreviations

ADL: Activities of daily living
LTC: Long-term care
SD: Standard deviation
